# Endoscopic Ultrasound-Guided Local Ablative Therapies for the Treatment of Pancreatic Neuroendocrine Tumors and Cystic Lesions: A Review of the Current Literature

**DOI:** 10.3390/jcm12093325

**Published:** 2023-05-07

**Authors:** Alexander M. Prete, Tamas A. Gonda

**Affiliations:** 1 Department of Medicine, New York University (NYU) Grossman School of Medicine, New York, NY 10016, USA; 2 Division of Gastroenterology and Hepatology, New York University (NYU) Langone Health, New York, NY 10016, USA

**Keywords:** EUS-guided local therapies, interventional EUS, radiofrequency ablation, ethanol ablation, chemical ablation, intratumoral drug delivery, pancreatic neuroendocrine tumors, pancreatic cystic neoplasms, pancreatobiliary disease

## Abstract

Since its emergence as a diagnostic modality in the 1980s, endoscopic ultrasound (EUS) has provided the clinician profound access to gastrointestinal organs to aid in the direct visualization, sampling, and subsequent identification of pancreatic pathology. In recent years, advancements in EUS as an interventional technique have promoted the use of local ablative therapies as a minimally invasive alternative to the surgical management of pancreatic neuroendocrine tumors (pNETs) and pancreatic cystic neoplasms (PCNs), especially for those deemed to be poor operative candidates. EUS-guided local therapies have demonstrated promising efficacy in addressing a spectrum of pancreatic neoplasms, while also balancing local adverse effects on healthy parenchyma. This article serves as a review of the current literature detailing the mechanisms, outcomes, complications, and limitations of EUS-guided local ablative therapies such as chemical ablation and radiofrequency ablation (RFA) for the treatment of pNETs and PCNs, as well as a discussion of future applications of EUS-guided techniques to address a broader scope of pancreatic pathology.

## 1. Introduction

Endoscopic ultrasound (EUS) emerged as a diagnostic modality approximately five decades ago and has since grown significantly in its utilization to aid in the identification of gastrointestinal (GI) pathology [1,2,3]. Given the proximity of the pancreas to the hollow organs of the GI tract, EUS offers excellent resolution of the pancreatic parenchyma, main duct, and its adjacent structures, including the common bile duct, portal and splenic veins, and mesenteric lymph nodes [1]. Therefore, EUS has become a reliable technique for the evaluation of pancreatobiliary disorders, demonstrating higher sensitivity in detecting early pancreatic tumors when compared to non-invasive imaging techniques such as positron-emission tomography (PET), computed tomography (CT), or transabdominal ultrasound [4]. Within the past decade, EUS has evolved from a purely diagnostic modality to an interventional technique, with new EUS-guided procedures showing great promise in addressing structural pathology of the pancreas.

Starting with the first human pilot study of EUS-guided ethanol ablation to treat pancreatic cystic neoplasms (PCNs) in 2005 [5], the field of EUS-guided local ablative therapy for pancreatic disease has shown subsequent expansion in both technique and therapeutic application. To date, ablative techniques are numerous and in various stages of clinical application, including chemical ablation (such as ethanol lavage and intratumoral chemotherapy delivery [6,7]), radiofrequency ablation, laser ablation, microwave ablation, and cryoablation therapy [8]. Though diverse in their mechanisms, these methods are unified in their minimally invasive approach, involving the use of ultrasound guidance to advance an electrode or needle tip into a target lesion while avoiding vascular or ductal structures [8]. These ablative techniques generate local necrosis of the target lesion while balancing potential adverse effects on healthy parenchyma (i.e., pancreatitis and pancreatic necrosis) and surrounding structures (i.e., portal venous thrombosis) [9].

EUS-guided ablative techniques have been applied to an ever-growing spectrum of pancreatic pathologies, chief among them neoplastic lesions. This includes both solid neoplasms, such as pancreatic neuroendocrine tumors (pNETs) and pancreatic ductal adenocarcinoma (PDAC), as well as cystic lesions. Traditionally, surgical management has been the definitive therapy of choice for neoplastic lesions that are symptomatic [9], malignant [10], or harboring malignant potential [11]. However, these procedures carry significant morbidity and mortality; depending on the malignant potential and individual risk, surgery may carry an unacceptable risk-to-benefit ratio [12]. EUS-guided local ablation has offered a minimally invasive therapeutic alternative to surgery [13]. Adding to its advantages, EUS-guided local ablative therapy has the potential to be conducted on an outpatient basis, resulting in reduced post-operative morbidity when compared to surgery [8].

Despite its growing appeal, the efficacy and safety of EUS-guided ablative therapies has been reported in the literature through a limited number of case reports and observational studies. To date, these therapies have yet to be compared vis-à-vis surgical management through a randomized controlled trial. While many ablative techniques remain in the experimental or pre-clinical phases of application, radiofrequency ablation (RFA) and chemical ablation with ethanol (EA) or chemotherapeutic substrate have been broadly reported in the literature, especially in the management of pNETs and PCNs. Thus, this article will serve as a review of selected studies reporting the use of EUS-guided RFA and chemical ablation in the treatment of pNETs and PCNs, providing an overview of therapeutic rationale, mechanisms, efficacy, safety, and pitfalls. Additionally, this article will briefly touch upon the future applications of EUS-guided local ablative therapy, including new ablative techniques and the growing pathologic scope of this exciting intervention.

## 2. Clinical Definitions and Classification of Pancreatic Lesions

### 2.1. Definitons and Classification of PCNs

PCNs are a common lesion, with an estimated prevalence of CT detectable asymptomatic cysts reported in the literature as 2.2% of the general population [14]. However, despite their frequency, cystic lesions of the pancreas constitute a heterogenous group of tumors that are classified according to their histopathologic features [15]. Broadly, the main groups of PCNs include serous cystic neoplasms (SCNs), mucinous cystic neoplasms (MCNs), and intraductal papillary mucinous neoplasms (IPMNs), with the latter two harboring the potential for malignant transformation [11]. Given this, current consensus guidelines suggest surveillance of MCNs and IPMNs with progression to surgical resection should high-risk or worrisome features develop [16]. However, the absence of widely accepted evidence-based guidelines for PCN management has posed the clinical challenge of weighing the risks of unnecessary surgery with the potential for untreated malignant evolution.

### 2.2. Definitons and Classification of pNETs

pNETs constitute a small percentage of all pancreatic tumors, comprising only 1.3% of all cancers that originate in the pancreas [17]. These tumors are generally classified as either functional or non-functional, depending on whether they are capable of releasing hormones that may produce symptoms in the afflicted individual. While 60–90% of pNETs are non-functional, functional pNETs produce hormones such as insulin, gastrin, glucagon, somatostatin, and vasoactive intestinal peptide, generating hallmark symptoms that usually lead to earlier clinical detection and subsequent management [18]. Insulinomas are the most common functional pNET and result from neoplastic growth of beta cells in the islets of Langerhans [18].

## 3. Clinical Rationale for EUS-Guided Local Ablative Therapy

### 3.1. Clinical Rationale for EUS-Guided Local Ablative Therapy of PCNs

Due to recent technologic advances in modern imaging modalities, the detection rate of PCNs has only increased, many of which constitute incidental findings [15]. In fact, it has been reported that incidental pancreatic cysts now make up nearly one-third of resected lesions seen in surgical practice [19]. EUS has become a potent tool to aid in pre-operative diagnosis and classification of either symptomatic or incidentally detected cystic lesions of the pancreas. International guidelines have distinguished features of potentially malignant cystic lesions (including cyst size, location, internal and capsular structure, and association with changes in the size or caliber of the main pancreatic duct (MPD) [20]), all of which can be detected using EUS. Additionally, EUS-guided fine needle aspiration (FNA) allows the clinician to sample intra-cystic fluid in real-time, with subsequent fluid cytology and analysis of carcinoembryonic antigen (CEA) levels to aid in the diagnosis of a mucinous neoplasm [21].

Should the diagnostic features of a mucinous PCN reveal high-risk for malignancy or high-grade dysplasia (HGD), definitive surgical management is deemed the gold standard therapy [22]. Unfortunately, in many instances, histologic diagnosis of HGD or carcinoma cannot be achieved preoperatively, and the clinician must make a management decision based on the radiographic and biochemical surrogate markers detailed above [23]. Thus, there is a growing clinical interest in exploring an effective, minimally invasive technique to treat pre-malignant lesions prior to their transformation to invasive carcinoma while also avoiding the significant perioperative morbidity and mortality of invasive surgery [24]. In patients with unilocular or oligolocular mucinous cysts without definite pancreatic mass who are poor operative candidates, ablation can be considered as a therapeutic option that avoids the safety concerns of invasive surgery while allowing for clinical management beyond conservative imaging surveillance [25].

### 3.2. Clinical Rationale for EUS-Guided Local Ablative Therapy of pNETs/Insulinomas

In general, pNETs exhibit heterogenous clinical behavior, spanning from incidental growths on imaging, to indolent and slow-growing masses, to aggressively metastatic lesions [26]. Recommendations for pNET management are provided by the National Comprehensive Cancer Network (NCCN) guidelines, which are largely based on tumor staging (including tumor size, nodal involvement, and presence of distal metastases) as well as histologic grading, which is generally defined by mitotic count and/or Ki-67 index on pathologic reporting [27]. Since tumor size remains an important correlate to malignant potential [28], all tumors greater than 2 cm are generally considered locally invasive and therefore warrant surgical resection along with regional lymphadenectomy [18].

However, given their unpredictable malignant potential, there remains great controversy surrounding the management of non-functional pNETs less than 2 cm in size, with some recommending a conservative “watch-and-wait” approach over invasive surgery [29]. Thus, there is a growing interest in EUS-guided ablation as a minimally invasive locoregional treatment modality that can balance the risks of overtreatment (i.e., surgical excision and its associated complications) and undertreatment (i.e., undetected malignancy in a patient undergoing conservative periodic surveillance) [30,31]. Additionally, since the prognosis of functional pNETs tends to be more favorable in comparison to non-functional tumors given their propensity to produce symptoms that contribute to earlier detection [32,33], EUS-guided ablation offers a therapeutic option for rapid symptom relief in those suffering from hormone over-production, especially when these patients may not be appropriate candidates for definitive cure with surgical intervention [34].

## 4. Mechanisms of Action for Select EUS-Guided Local Ablative Therapies

In 1992, the first case of EUS-guided FNA of a pancreatic head lesion was reported by Vilmann et al. [35], signifying a revolutionary step in the clinical diagnosis and staging of GI pathology. Through the decades, the techniques of EUS-guided FNA have been modified to serve an interventional role, resulting in the field of EUS-guided local ablative therapy. Although several ablative therapies have been described in the literature, this portion of this review article will largely focus on the mechanisms of the two most widely used techniques: chemical ablation (including EA and intratumoral drug delivery) and RFA.

### 4.1. Mechanisms of EUS-Guided EA

The use of ethanol as a chemical ablative substrate has a rich history in clinical medicine, spanning the spectrum from thyroid cyst therapy [36] to alcohol septal ablation for hypertrophic obstructive cardiomyopathy [37]. In the field of GI specifically, the efficacy of percutaneous ethanol lavage of cystic lesions in solid organs such as the liver [38] and spleen [39] has been reported for decades. Given the success of these therapies with evidence of significant reduction in cyst volume even after a single session [38], these techniques have evolved to address pancreatic pathology through endoscopic intervention.

In 2005, Gan et al. reported the first study in a human model that utilized EUS-guided ethanol ablation to treat pancreatic cystic lesions [5], demonstrating both the efficacy and clinical feasibility of this intervention. Ethanol’s popularity as a chemical ablative agent has since persisted due to its low cost, abundant availability, and rapid-acting ablative capacity [15]. Ethanol is a short-chain alcohol that, at high concentrations, solubilizes the cell membrane and alters protein tertiary structure [40]. Thus, by injecting the toxic substrate into a cystic cavity or neoplastic lesion, ethanol promotes cellular death through a combination of membrane lysis, protein denaturation, and vascular occlusion [41]. Additionally, cytotoxicity of ethanol is enhanced through mitochondrial injury and disruption of intracellular signal transduction [42]. Together, these mechanisms generate tissue necrosis in target lesions, which can be localized to pathologic tissue under ultrasound guidance.

### 4.2. Mechanisms of EUS-Guided Intratumoral Drug Delivery

Intratumoral injection of chemotherapeutic or other biological antitumor agents has previously been described in the treatment of conditions such as endobronchial non-small cell lung tumors [43] and pediatric brain cancers [44]. Recently, EUS has made possible the local delivery of chemotherapeutic agents (namely, paclitaxel) to treat cystic tumors of the pancreas while mitigating systemic side effects [6,7]. Paclitaxel is a hydrophobic and viscous chemotherapeutic agent, thereby exerting a long-lasting antineoplastic effect on a closed, cystic cavity with low possibility of leakage into surrounding healthy tissue [6]. Oh et al. [7] initially reported the safety, feasibility, and efficacy of EUS-guided ethanol lavage with paclitaxel injection for PCNs through a prospective pilot study. They described a potential synergistic effect between the two chemical agents, with primary distortion of cystic epithelium by ethanol followed by secondary antitumor effect from microtubule inhibition with paclitaxel. Additionally, there is evidence that EUS-guided ethanol lavage with paclitaxel can alter mutant DNA (including KRAS mutations) present in pre-treated cystic fluid, potentially interrupting progression to malignancy [45].

### 4.3. Mechanisms of EUS-Guided RFA

RFA as an interventional technique has previously shown efficacy in the palliative treatment of solid, unresectable tumors throughout the body, including the lungs [46], bone [47], prostate [48], and kidneys [49]. The safety of EUS-guided RFA of the pancreatic head was first shown by Gaidhane et al. [50] in 2012 utilizing a porcine model, which demonstrated the targeted potential of RFA therapy to generate discrete areas of necrosis while minimizing focal acute pancreatitis in healthy tissue. Since this initial study, the use of EUS-guided RFA has expanded to include human subjects seeking therapy for a spectrum of pancreatic neoplasms, including PDAC [51].

RFA harnesses the antitumor effects of hyperthermia, inducing cellular protein denaturation and subsequent coagulative necrosis [52]. Since human cells cannot typically withstand temperatures above 50 °C, the use of high-frequency alternating current (usually 200–1200 kHz frequency) delivered via an ultrasound-guided electrode leads to local agitation and friction with subsequent heat generation to temperatures as high as 90 °C [51]. Since maximum heat is generated in the vicinity closest to the electrode, this leads to decreased tumor bulk while minimizing side effects on healthy adjacent parenchyma [53]. Additionally, RFA is believed to produce cellular debris that promotes antigen presentation to lymphocytes, thereby enhancing antitumor effect by stimulating tumor-specific T cells and activating systemic immunity [54].

## 5. Clinical Applications of EUS-Guided Ablative Therapies for Pancreatic Pathology: A Summary of Reviewed Studies

As the technical scope of EUS-guided ablation has expanded, so has the spectrum of pathology addressed by this minimally invasive intervention. The focus of this literature review will be the use of EUS-guided chemical ablation and RFA for the therapy of PCNs and pNETs, with a summary of outcomes and complications of select studies in the sections to follow.

### 5.1. EUS-Guided EA for the Treatment of PCNs

The efficacy and safety of EUS-guided EA for the treatment of PCNs has been reported in several observational trials [5,24,55,56,57,58] and a randomized trial [59] (Table 1). Gan et al. [5] published the first pilot study of EUS-guided EA of cystic lesions at Massachusetts General Hospital in 2005. In this prospective, single-center study, 25 asymptomatic patients with image-confirmed pancreatic cystic lesions (including MCNs, IPMNs, SCNs, and one pseudocyst) were selected to undergo EUS-guided cyst aspiration followed by ethanol lavage. The results were promising, revealing complete cyst resolution in 35% of participants at one-year follow-up. When assessing outcomes according to pre-procedural diagnosis, 62.5% of MCNs were completely resolved at follow-up while 100% of IPMNs persisted despite therapy. Importantly, no documented adverse events were noted for 72 h post-procedure. Thus, in addition to proving the technical feasibility of this procedure, Gan et al. also showed that EUS-guided EA of PCNs is a safe intervention with a theoretically low risk of precipitating pancreatitis.

In 2009, DeWitt et al. [59] designed a prospective, multicenter, double-blind randomized controlled trial to compare the efficacy of EUS-guided ablation with ethanol to that of saline lavage in the treatment of a spectrum of pancreatic cysts (including MCNs, IPMNs, SCNs, and pseudocysts). The results revealed that lavage with 80% ethanol resulted in significant reduction in cyst size when compared to injection of saline solution alone, and that one or more sessions of EA led to complete cyst resolution in 33% of patients who completed follow-up. Evidence for the ablative potential of ethanol substrate was furthered by histopathologic examination: four patients in the study later underwent surgical resection of their mucinous cysts, revealing 0% cyst epithelial ablation in the participant treated with saline lavage alone versus 50–100% observed in those who received one or two sessions of EUS-guided EA. Although patients were randomized to receive either one or two sessions of EA, the study was underpowered to reveal any benefit in cyst reduction when comparing the two groups. In contrast to Gan et al., complications were observed in this study, with 12–16% of patients experiencing abdominal pain within one week of the procedure and two patients developing acute pancreatitis due to extravasation of ethanol from the cyst into adjacent parenchyma.

In 2010, the same group conducted a prospective cohort study that provided long-term follow-up of cysts that were successfully ablated with one or two sessions of EA in their original study [55]. Of the 12 patients in the initial study who experienced radiographically confirmed resolution of their PCNs after EUS-guided ablation, 9 participants underwent repeat CT scan at a median of 26 months after documentation of complete cyst ablation, demonstrating absence of recurrence in all patients. This study supported the long-term durability of cyst ablation using EUS-guided EA, revealing the potential for this intervention to “cure” individuals of their PCNs.

While these prior studies demonstrated the feasibility, safety, and durability of EUS-guided EA for PCNs, there remained a question regarding the potential therapeutic benefit of conducting multiple sessions of ethanol lavage in comparison to a single treatment course. Thus, in 2011, DiMaio et al. [56] conducted a retrospective review of 13 patients with asymptomatic, benign-appearing IPMNs who underwent two or more sessions of EA for cyst ablation given their status as poor surgical candidates. They observed a significantly greater decrease in cyst diameter and surface area after two sessions of EUS-guided EA in comparison to a single session. Although image-confirmed cyst resolution did not occur in any patient after a single EA session, it occurred in 5 patients (38% of participants) after their second course of EA. Additionally, the group noted only minor abdominal pain after the first and second EA sessions in a single patient, further supporting the safety of this intervention.

In 2012, Caillol et al. [24] sought to understand the efficacy of EUS-guided ablation of MCNs specifically, conducting a bi-center prospective cohort study of 13 patients who received EA for treatment of their mucinous cysts given their contraindications to surgery (including heart failure, hypertension, and recent cancer). At a follow-up of 26 months post-procedure, 85% of patients had complete cyst ablation on imaging, a high success rate that can likely be attributed to the low sample size and strict inclusion criteria (as ablated cysts were small in diameter and lacked septation). Later in 2016, Park et al. [58] completed a clinical study of 91 participants with unilocular or oligolocular pancreatic cysts (including an overwhelming majority of SCNs and indeterminate lesions) treated with a single session of EUS-guided EA. Although overall treatment response was high with 45% of participants experiencing complete resolution at 40-month follow-up, the success rate varied significantly according to pre-procedural cyst classification: while 50% of patients with MCNs achieved cyst resolution, only 11% of IPMNs were responsive to EA. The authors speculated that this was likely multi-factorial, including the presence of a complex papillary growth pattern in IPMNs as well as communication with the MPD (as the treated cysts were branch duct IPMNs) that may have collectively diminished the ablative effects of ethanol. In the absence of concrete evidence to support these speculations, the authors concluded that further investigations are required to determine how cystic fluid parameters can function as surrogate markers for predicting the success of EUS-guided EA for PCNs.

While these studies support the promising therapeutic efficacy of EUS-guided EA for PCNs, this is not the case for all trials. In 2015, Gómez et al. [57] conducted a single-center, prospective pilot study of 23 patients with cystic lesions (a majority of which were MCNs or IPMNs) treated with EUS-guided EA, reporting less than 80% cyst size reduction at 6-month follow-up in 10 patients and even a 73% increase in cyst volume in one treated patient. Additionally, surveillance imaging conducted at annual intervals post-procedure revealed an increase in cyst volume in 9 treated participants. Complete cyst resolution occurred only in two patients, one of whom was diagnosed with a presumed unilocular IPMN; otherwise, 93.3% of treated IPMNs persisted on follow-up imaging. When comparing participants who achieved 80% or greater initial reduction in cyst volume to those with less than 80% reduction, the authors reported no significant differences regarding patient demographics, cyst characteristics (including initial cyst volume, cyst CEA concentrations, or number of cystic locules), or ethanol concentration between the study groups. However, the authors did report that cysts presumed to be non-mucinous in composition experienced a greater reduction in size compared to those presumed to be mucinous, supporting the findings reported by Park et al. In terms of safety, only two participants experienced complications within 24 h of treatment, including one case of pancreatitis that resulted in hospitalization. Unfortunately, one patient with presumed IPMN was diagnosed with PDAC 41 months following EUS-guided EA, with the cancer likely arising from the treated cyst despite an initial observed reduction in cyst volume of 69% after endoscopic intervention. While median radiographic follow-up in this study was cited at 37.3 months, recent large studies of patients with branch-duct IPMNs have revealed a 5-year incidence rate of pancreatic malignancy of 3.3%, which increases to 15% at 15 years post-diagnosis [60]. Since the risk for malignant degeneration of IPMNs is elevated compared to the general population even after 5 years of surveillance [60], the follow-up period of this study (as well as the other studies reviewed in this section) was likely too brief to capture the cumulative risk of malignant conversion in the study population. This unfortunate outcome therefore highlights the need for sustained follow-up of PCNs with malignant potential treated with EA to effectively monitor for the clinical goal of preventing malignant conversion and progression.

### 5.2. EUS-Guided Intratumoral Drug Delivery for the Treatment of PCNs

The efficacy of EUS-guided intratumoral drug delivery for the treatment of PCNs has been reported in several observational trials [6,7,61,62,63] and a randomized trial [64] (Table 2). Oh et al. [7] first described the feasibility and safety of EUS-guided paclitaxel injection following EA of 14 PCNs at a single center in 2008. This procedure was safely performed in all but one patient, with only one reported case of mild acute pancreatitis that resolved with supportive care. Additionally, at mean follow-up of 9 months, complete resolution was observed in 11 patients, with the authors reporting better treatment response in smaller cysts less than 3 mL in volume. The same group subsequently performed a larger prospective study of 52 patients with PCNs in 2011 that observed the outcomes of a similar treatment algorithm of EA followed by paclitaxel injection [6]. At mean follow-up of 21.7 months, complete resolution was achieved in 29 patients, with univariate analysis describing smaller EUS-measured cyst diameter and volume as predictors of treatment success. Although this study did not reveal an association between the presence of cystic septa and the likelihood of post-treatment resolution, the same group performed a 2009 study of 10 patients with oligo-septated PCNs who underwent EUS-guided EA followed by paclitaxel injection [61]. While complete resolution was observed in 6 patients, post-operative evaluation of persistent cysts resected from two patients revealed remnant neoplastic epithelial lining in missed locules, suggesting that cyst morphology may play an important role in proper candidate selection for EUS-guided chemical ablation.

In 2017, Choi et al. [62] investigated the long-term durability of EUS-guided chemical ablation of PCNs with ethanol and paclitaxel by conducting a single-center, prospective study of 164 patients with median follow-up of one- and 6-years duration. At one-year follow-up, the authors reported complete cyst resolution in 72.2% of participants, with subsequent multivariate analysis revealing cyst diameter less than 35 mm and absence of septation as significant predictors of complete response. Interestingly, cystic lesions presumed to be IPMNs based on pre-procedural fluid analysis displayed the lowest rate of complete resolution (only 50%, compared to 76.1% of MCNs), supporting the results previously reported by Park et al. [58] suggesting that therapy of an IPMN may not be the optimal indication for EUS-guided chemical ablation. Of the 114 patients with complete cyst resolution at one-year post-procedure, radiologic cyst recurrence was noted in only 2 patients at a median follow-up of 72 months with no reported cases of malignancy during this time. Given complete cyst resolution in 98.3% of participants at long-term follow-up, the authors concluded that EUS-guided chemical ablation of PCNs with ethanol and paclitaxel induces a durable treatment response; however, the presence of recurrence in a small number of patients indicates the need for surveillance imaging post-procedure.

In 2017, Kim et al. [63] sought to evaluate the sonographic and cytological changes associated with EUS-guided PCN ablation, designing a prospective, single-center study of 36 patients with benign-appearing cysts who received therapy with ethanol alone (8 patients) or with a combination of ethanol and paclitaxel (28 patients). Although not specifically designed to compare these two chemical ablative regimens, this study revealed that the combination of ethanol and paclitaxel increased the quantity but decreased the quality of cystic DNA after EUS-guided ablation. The authors owed this finding to likely increased epithelial cell turnover after ablation, as well as the potential influx of inflammatory cells into cystic fluid as a response to one or both ablative agents. These findings supported a previous observation that EUS-guided chemical ablation may eliminate mutant cystic DNA [45].

To determine whether alcohol is required for effective PCN ablation, Moyer et al. [64] conducted a single-center, double-blind, randomized controlled trial of 39 patients with mucinous-type pancreatic cysts who first received EUS-guided lavage with either ethanol or normal saline, followed by an infusion of paclitaxel and gemcitabine. Despite a previously postulated synergistic effect between the two substrates, there was no statistically significant difference in complete ablation rates at one-year follow-up between those who underwent alcohol-free chemical ablation versus those who first received ethanol lavage. Additionally, no serious adverse events were observed in the alcohol-free group, while one case of acute pancreatitis was reported in the ethanol arm. These results suggest that alcohol is not required for successful ablation if an effective antitumor chemical agent is used in its place, and that alcohol’s addition to a chemotherapeutic substrate may incur a higher complication rate. Thus, the removal of ethanol from EUS-guided chemotherapeutic regimens may preserve clinical efficacy while mitigating side effects.

While these prior studies sought to measure the efficacy and durability of EUS-guided intratumoral drug delivery based on post-procedural radiographic cyst resolution, An et al. [65] recently reported the histopathologic characteristics of 12 surgically resected PCNs following EUS-guided local ablation with ethanol and/or paclitaxel. Based on pre-treatment imaging, a majority (84%) of these lesions were believed to be MCNs, with a mean cyst size that was similar pre- and post-procedure. Therefore, all 12 participants underwent surgical resection at a median of 18 months following initial ablation, with subsequent pathologic examination revealing 8 cases (67%) with either complete absence of or <5% residual lining epithelia. Based on these results, the authors concluded that, when compared to untreated MCNs, pancreatic cysts treated with EUS-guided local ablation may display wider areas of cystic walls free from covering lining epithelium. Although the clinical implications of the study cannot be extrapolated given the small sample size, these results suggest that EUS-guided chemical ablation with ethanol and/or paclitaxel likely induces histologic cystic changes on the tissue level that can be present even in the absence of a complete or partial radiographic response.

### 5.3. EUS-Guided RFA for the Treatment of PCNs

Although less studied than EA, the efficacy of EUS-guided RFA for the treatment of PCNs has been reported in several observational trials [11,66,67,68] (Table 3). In 2015, Pai et al. [11] designed the first multicenter pilot study that investigated the safety and feasibility of using EUS-guided RFA to treat PCNs in the head of the pancreas of 6 patients. Follow-up imaging was obtained 3–6 months post-procedure, which revealed complete cyst resolution in 2 patients and partial response with 48.4% reduction in cyst size in 3 patients. In terms of safety, only 2 patients experienced mild abdominal pain that resolved within 3 days post-procedure, but there were no episodes of pancreatitis, perforation, or bleeding.

In 2019, Barthet et al. [66] designed a multicenter, prospective study of 17 patients with either an IPMN or a MCN who were treated with EUS-guided RFA, a new procedure for two of the sites included in the investigation. The primary objective of this study was to assess for procedural safety, with a secondary outcome of observing antitumor effect. Due to post-procedural complications observed in the first two patients (one of whom was being treated for a pNET, not a PCN), the group introduced a procedural prophylaxis of rectal diclofenac and antibiotic coverage with amoxicillin-clavulanic acid for all subsequent patients. Overall, this resulted in improved outcomes, with no additional serious complications of pancreatitis, perforations, or infections in those receiving treatment for their PCNs. The procedure also proved to be efficacious, with a complete response observed in 8 patients at six-month follow-up that increased to 11 patients at one-year. Interestingly, the authors attributed this increased response at one-year to the immunostimulatory effects of residual tumoral antigen produced through RFA-induced necrosis and cell death.

In 2022, Younis et al. [67] conducted a prospective single-center study of 5 patients with either an IPMN or a MCN who were treated with EUS-guided RFA after prophylaxis with the same regimen described in Barthet et al. Results revealed complete response in 3 patients and only 3 cases of relatively minor complications. Taken together, these two studies support the safety and technical feasibility of EUS-guided RFA for the treatment of mucinous cysts, although their small sample sizes, short follow-up, and lack of a control arm limit their clinical impact.

Departing from these studies, Oh et al. [68] sought to evaluate the feasibility and safety of EUS-guided RFA for the treatment of SCNs in particular, designing a prospective study of 13 patients who underwent single or multiple sessions of RFA intervention with follow-up imaging approximately 9 months post-procedure. Although no participants had complete cyst resolution, partial response with cystic volume reduction by 66% was observed in 8 patients, along with an acceptable adverse event rate of one case of mild, self-resolving abdominal pain. The authors speculated that the seemingly lower efficacy observed in their study was due to the complex morphology of the treated cysts, as they all had a honeycomb appearance with multiple septations that may have prevented heat delivery into multiple locules.

### 5.4. EUS-Guided EA for the Treatment of pNETs

The feasibility, efficacy, and safety of EUS-guided local EA for the treatment of pNETs (especially insulinomas) has been reported in several observational trials [31,69,70,71] (Table 4). In 2012, Levy et al. [69] performed the first retrospective study of 5 patients with either sporadic or multiple endocrine neoplasia 1-associated insulinomas who underwent two or more sessions of EUS-guided chemical ablation with 95–99% ethanol. At median follow-up of 13 months following their last session, 3 patients reported complete resolution of their hypoglycemic symptoms (although one patient was still taking daily diazoxide), while the other 2 reported marked improvement in the frequency and severity of their symptoms. Additionally, there were no intraprocedural or postprocedural complications observed in these participants, thereby supporting the safety of this intervention. Nonetheless, the study was limited by its small sample size, its absence of standardized follow-up imaging to monitor for treatment-induced morphologic response, and its retrospective, uncontrolled design.

To assess the feasibility and safety of this intervention, Park et al. [31] performed a retrospective analysis of a prospectively collected database of 11 patients with 14 pNETs (4 insulinomas and 10 non-functional pNETs) who were treated with one or more sessions of EUS-guided EA. Of the patients who underwent a single treatment session, 3-month radiographic follow-up revealed complete resolution in 7 tumors; three tumors that had not resolved were subjected to re-ablation, after which the total number of tumors with complete response was increased to 8 (or, 61.5% of all tumors at follow-up). Additionally, both patients who received treatment for their insulinomas reported complete resolution of hypoglycemic symptoms at follow-up. Based on these results, the authors deemed EUS-guided EA a technically feasible intervention for the treatment of pNETs specifically in those who refuse surgery or who are deemed to be poor surgical candidates. In terms of safety, 3 patients experienced acute pancreatitis immediately post-procedure; one of these patients was subsequently found to have a MPD stricture requiring stent placement. Interestingly, all patients who developed pancreatitis received more than 2 mL of ethanol in a single session, suggesting a potential dose-dependent response to ethanol-related toxicity on local healthy parenchyma.

In an effort to mitigate these complications, Choi et al. [70] designed a prospective study of 33 patients with 40 pathologically confirmed pNETs who underwent one or more sessions of EUS-guided chemical ablation with a mixture of ethanol and lipiodol. When combined with ethanol, lipiodol (an iodized poppy seed oil) had previously shown promise as an ablative agent in the chemoembolization of unresectable hepatocellular carcinoma [72,73], working to occlude microvasculature while also serving as a contrast agent for detection of drug delivery. Compared to Park et al., Choi et al. reported a comparatively lower rate of adverse events (3.6%); the authors attributed this phenomenon to the presence of the fatty acid lipiodol, which enhanced chemical retention in the tumor without leakage into surrounding parenchyma. Furthermore, lipiodol retention within the tumor following EUS-guided EA served as a significant predictor of complete ablation (*p* = 0.004), thereby supporting the use of post-procedural lipiodol retention seen on CT or fluoroscopic imaging as an early predictor of interventional success.

More recently, Matsumoto et al. [71] sought to investigate the efficacy of early EUS-guided ethanol reinjection for patients with pNETs, designing a prospective pilot study of 5 patients with small pNETs who all underwent initial EA with subsequent contrast-enhanced CT imaging conducted 3 days post-procedure; for the 3 patients with residual enhancement, an additional session of EA was conducted while the patient was still hospitalized. Results revealed complete ablation without recurrence at one-year follow-up in 4 patients (80%), and there were no reported complications in those who received early reinjection. Although this study supported the safety and feasibility of this protocol, the absence of a large sample population, the lack of a comparative group, and the short follow-up duration limited its clinical impact.

### 5.5. EUS-Guided RFA for the Treatment of pNETs

There exists a robust and rapidly growing body of literature reporting the feasibility, safety, and efficacy of EUS-guided RFA for the treatment of pNETs, with a review of selected studies summarized below [11,66,74,75,76,77] (Table 5). Pai et al. [11] performed the first multicenter prospective pilot study assessing the feasibility of EUS-guided RFA for the treatment of 2 patients with non-functional NETs of the pancreatic head. On follow-up cross sectional imaging, a change in the tumor vascularity was noted in one patient, while two sessions of RFA in the second patient resulted in an area of central tumor necrosis. Importantly, no adverse events were noted in these patients, demonstrating the safety of the procedure. Several years later in 2019, Barthet et al. [66] conducted a larger prospective multicenter trial of 12 patients with 14 non-functional pNETs treated with EUS-guided RFA, reporting complete radiographic resolution at one-year follow-up in 85.7% of tumors. Two serious complications were noted in this study: one case of acute pancreatitis with an area of infected necrosis, which was observed in a patient who did not receive prophylaxis of amoxicillin-clavulanic acid and rectal diclofenac; the second was a case of MPD stenosis in a patient who did receive prophylaxis, requiring treatment with endoscopic stenting. Taken together, these studies supported the efficacy and favorable safety profile of using EUS-guided RFA to treat pre-malignant non-functional pNETs.

Oleinikov et al. [74] conducted an even larger retrospective multicenter study of 18 adult patients with 27 neuroendocrine lesions (including insulinomas and non-functional pNETs) treated with EUS-guided RFA. In terms of technical feasibility, 96% of tumors were successfully ablated based on EUS visualization immediately post-procedure, while one tumor experienced incomplete ablation due to its proximity to the MPD. Compared to prior studies, Oleinikov et al. included 7 patients with functional pNETs, thereby evaluating the efficacy of EUS-guided RFA in the treatment of symptoms related to hormone over-production. The authors reported that all study participants with insulinomas achieved immediate symptom relief and euglycemia within one hour of the procedure. Additionally, this treatment response was durable, as no symptom recurrence was noted by any of the patients at a mean follow-up of 9.7 months. Two cases of mild acute pancreatitis were noted and resolved with conservative treatment within an average of 3 days. Importantly, this study included 5 patients who were initially offered serial surveillance imaging of their incidentally diagnosed, small, and asymptomatic non-functional pNETs, but refused due to the emotional burden of a “wait and see” approach. Thus, while prior studies mainly included participants who were poor operative candidates, Oleinikov et al. demonstrated that EUS-guided RFA is a safe and feasible approach for those seeking a more definitive alternative to surveillance for the treatment of their incidental pNETs.

Marx et al. [75] conducted a retrospective review of EUS-guided RFA specifically for the treatment of insulinomas at two tertiary referral centers, reporting the periprocedural safety and outcomes for 7 patients with radiographic follow-up via magnetic resonance imaging (MRI)/CT. Prior to the procedure, all participants endorsed episodic symptomatic hypoglycemia with significant impact on quality of life that necessitated frequent hospitalization. However, post-procedure, all patients reported immediate symptom relief accompanied by euglycemia that persisted throughout follow-up, with complete tumor resolution observed in 6 patients at 12–18 months post-procedure. However, safety was a concern in this study, with one patient developing acute pancreatitis despite preventive stent placement due to the tumor’s proximity to the MPD, while another developed an area of coagulative necrosis because of the tumor’s proximity to the superior mesenteric vein. Unfortunately, a frail elder patient was found to have a retro-gastric collection two weeks post-procedure, which ultimately resulted in her death prior to evaluation for treatment response.

The same group [76] conducted a much larger multicenter retrospective review of 27 patients with non-functional pNETs, reporting excellent efficacy with complete resolution of 93% of tumors after one or more sessions of EUS-guided RFA at a mean follow-up of 15.7 months. Relevant complications included three cases of acute pancreatitis, one of which resulted in pseudocyst formation and two of which required cystogastrostomy for drainage of retro-gastric/retro-splenic collections. The authors could not identify a single unequivocal risk factor for the development of pancreatitis, although they suggested the possibility of exploring a step-up approach for larger lesions to reduce adverse events generated by single sessions aimed at complete ablation.

Most recently, Figueiredo et al. [77] conducted a large, prospective multicenter study that evaluated the safety and clinical efficacy of EUS-guided RFA of 29 patients with a spectrum of 35 pancreatic and peripancreatic tumors, including 10 non-functional pNETs, 13 insulinomas, 1 PDAC, and 11 intra-pancreatic and extra-pancreatic metastatic lesions (largely arising from metastatic lung and renal carcinoma). Of the 15 pNETs with 6-month follow-up, 73.3% showed a significant response to intervention with either complete necrosis or greater than 50% size reduction on imaging. In terms of clinical response for those receiving therapy of their functional pNETs, 100% of cases resulted in immediate resolution of hypoglycemia post-procedure, with no symptom recurrence during median follow-up of 9.5 months. Thus, Figueiredo et al. concluded that functional pNETs were seemingly the best indication for EUS-guided RFA therapy, reporting high efficacy in symptom reduction along with an acceptable safety profile.

## 6. Complications of EUS-Guided Local Ablative Therapies

As detailed in the above studies, EUS-guided local ablative therapies are associated with a spectrum of mild to severe adverse events. Complications may arise from the endoscopic technique, including perforation, infection, and hemorrhage [66]. Additionally, treatment of cystic structures with chemical substrate can lead to peri-cystic spillage and intra-cystic hemorrhage [62,63]. While ultrasound-guidance allows for targeted delivery of ablative substrates to pathologic tissue, complications can also arise when normal parenchyma is damaged. Acute pancreatitis is a commonly described complication that is largely responsive to supportive treatment; however, progression to pancreatic necrosis [66] or MPD stenosis requiring stent placement [31,62,66] has been described. In particular, EUS-guided EA of branch duct IPMNs raises concern for extravasation given the presence of a widely patent communication with the adjacent ductal system, thereby increasing the risk of complications such as MPD stenosis [5]; as a result, some consensus guidelines have considered the presence of communicating IPMNs as a contraindication to EUS-guided EA [20]. Pancreatic pseudocyst formation has been described as a complication of EUS-guided RFA [76], which can increase the risk of future infection, hemorrhage, rupture, or ductal disruption. Finally, given the proximity of the pancreas to the portal venous system, EUS-guided treatment of cysts close to venous structures can lead to chemical extravasation and subsequent portal vein thrombosis [62] or splenic vein obstruction [6,62].

## 7. Limitations in EUS-Guided Local Ablative Therapies for Pancreatic Pathology

Despite the promising results of EUS-guided local ablative therapies as detailed in the above studies, there are important considerations that limit the quality of evidence in the current literature. Several of the aforementioned studies suffer from a limited, unrandomized sample population, thereby reducing the generalizability and clinical impact of their reported results. Additionally, many studies lack the long-term follow-up that is necessary to adequately monitor for PCN or pNET resolution post-procedure. As in Gómez et al. [57], there exists the possibility of malignant progression despite initial EUS-guided therapy, which may not be observed within the limited follow-up reported in the current literature. In fact, for PCNs of malignant potential in particular, some guidelines recommend surveillance cross-sectional imaging at 6-month intervals for the first year post-procedure, followed by annual imaging until patient co-morbidities and age limit the survival benefit of surveillance [25]; unfortunately, the vast majority of the above studies do not provide this duration of follow-up, and therefore the results may overstate the efficacy of ablation in the short term. Nearly all studies are observational in nature and are thereby limited by the absence of a control arm, which would be useful for comparing outcomes among those who opt for surgical management or conservative surveillance over EUS-guided local therapy. Finally, treatment response in the literature is typically monitored via interval change in tumor dimensions on cross-sectional imaging. Since this method does not necessarily confirm complete histopathologic ablation on the tissue level, it may be inadequate to assess the true efficacy of EUS-guided intervention in generating complete tumor necrosis and regression.

As a technique, EUS-guided ablation is limited in its therapeutic scope by important technical and safety considerations. Firstly, although procedural side effects are typically manageable, there remains a risk for serious complications, including MPD stenosis, pancreatic necrosis, bowel perforation, and even death. More data are necessary to assess how clinician expertise and institutional volume affect the frequency of these observed complications. Secondly, EUS-guided local ablation is limited in its ability to definitively address advanced local and metastatic disease, as extensive lymph node dissection is not yet technically feasible with endoscopy. Finally, in comparison to surgical intervention, the absence of resected specimen that can be assessed for tumor margins limits the extent to which EUS-guided ablative therapy can be considered as a form of definitive management for neoplastic pathology.

While many of the above studies sought to investigate the safety, technical feasibility, and efficacy of EUS-guided local ablative therapies, their results raised important inquiries for future clinical research. There remains a question of the efficacy of EUS-guided alcohol ablation specifically for the indication of pancreatic IPMNs, with evidence suggesting a decreased propensity for cyst reduction following intervention when compared to outcomes for MCN ablation [58,62]. Perhaps more importantly, the malignant progression of a treated IPMN observed in Gómez et al. [57] highlights the notion that size reduction does not necessarily correlate with decreased risk of future malignancy [25]; therefore, post-procedural surveillance remains an important consideration for future investigation to determine the long-term outcomes and clinical utility of EUS-guided ablation for IPMNs. Together, these results have led some international consensus guidelines to avoid recommending EUS-guided EA for the treatment of IPMNs outside of the context of a controlled research protocol until future data is available [20]. Additionally, data is limited concerning the proper course of action for therapeutic intervention of cysts in close proximity to the MPD, with some studies reporting prophylactic placement of an endoscopic stent to prevent acute pancreatitis post-treatment [75]. Finally, while some studies reported the apparent effects of cyst morphology (especially the presence of septations [61,68]) on treatment efficacy, comparative trials are necessary to understand the specific clinical features that predict treatment success to optimize candidate selection.

## 8. New Horizons: Future Applications of EUS-Guided Local Ablation

While this study has largely focused on the literature of EUS-guided chemical ablation and RFA for the treatment of PCNs and pNETs, there are exciting new horizons for the technical and therapeutic scope of this minimally invasive intervention.

### 8.1. New Ablative Techniques to Address Pancreatic Pathology

Apart from RFA and chemical ablation with ethanol and/or paclitaxel/gemcitabine, new ablative techniques are currently being investigated and are in various stages of clinical application. In addition to the intratumoral injection of chemotherapeutic agents, chemical ablation with the sclerosant agent lauromacrogol has recently shown efficacy in the local therapy of PCNs [78]. By inducing severe local inflammation and intramural fibrosis of vascular structures, lauromacrogol has previously been utilized in the mechanical obliteration of gastric varices in patients with liver cirrhosis [79]. It has also been applied to the clinical treatment of hepatic cysts [80] and the experimental therapy of endometrial cysts in an animal model [81]. In 2017, Linghu et al. [78] was the first group to assess the safety and efficacy of EUS-guided PCN ablation with lauromacrogol in 29 patients with imaging follow-up at a mean of 9 months post-procedure. The authors reported complete resolution in 37.9% of participants, with mild procedural complications occurring in 3 patients. Given the absence of severe adverse events, the authors concluded that EUS-guided local ablation with lauromacrogol is a safe intervention, with the potential added benefit of providing intra- and post-operative pain relief due to its mild anesthetic effect. The same group [82] conducted a study of the long-term outcomes of EUS-guided lauromacrogol ablation in 55 patients with median follow-up of 15 months, reporting a similar rate of complete cyst resolution of 47.3%. Despite its promising safety profile, the resolution rate noted in the aforementioned studies appears to be similar to the lower rates of effective ablation seen with ethanol [25,83], and therefore chemical ablation with ethanol or chemotherapeutic substrate largely remains the preferred technique. Additionally, both studies specifically excluded patients with IPMNs, thereby limiting the therapeutic scope of this modality.

The feasibility of EUS-guided laser ablation (LA) of pancreatic tissue with the neodymium-doped yttrium aluminum garnet laser was initially demonstrated in a pig model in 2010, where it was shown to induce localized tissue necrosis with the advantage of great precision [84]. By utilizing a finer needle, EUS-guided LA has become an attractive option for the treatment of lesions in high-risk areas or locations that are more technically difficult to access [85]. In 2018, Di Matteo et al. [86] proved the feasibility of this intervention in 9 patients with unresectable PDAC, demonstrating technical success in all patients without adverse events. Since its introduction to clinical application, EUS-guided LA has undergone changes in technical design, including the development of cylindrical interstitial laser ablation (CILA). This technique uses a diffusing application to help ablate tissue in a circular shape, thereby minimizing thermal damage to healthy parenchyma [87]. Although not in clinical use, EUS-guided CILA was demonstrated to be technically feasible in a porcine model of locally advanced PDAC, generating large areas of uniform ablation without significant complications [88].

Microwave ablation (MWA) is based on the production of frictional heat through the oscillation of dipole molecules, thereby inducing consistent and homogenous energy delivery to a discrete area of tissue [89]. Despite several studies demonstrating the safety and feasibility of percutaneous MWA on locally advanced pancreatic head cancer [90,91], EUS-guided MWA remains largely in the experimental phase of investigation, with one case report reporting technical success of the intervention in a poor surgical candidate with an unresectable neuroendocrine tumor of the pancreas [89].

Finally, EUS-guided cryoablation, often used in combination with RFA, was initially shown to be technically feasible in a porcine pancreas in 2008 [92], with subsequent studies demonstrating its safety and efficacy in patients with local advanced pancreatic cancer [93].

### 8.2. Growing Pathologic Scope of EUS-Guided Ablative Application

EUS-guided local ablation has been shown to be a technically feasible therapeutic option for patients with unresectable PDAC [94,95]. PDAC generally has a poor prognosis, with a 5-year overall survival of approximately 9% [96]. In large part, this is because most patients present with locally advanced or metastatic disease at the time of diagnosis, which limits therapeutic options including surgery, chemotherapy, or chemoradiation. EUS-guided local ablative therapies (namely, RFA) have emerged as promising treatment alternatives for PDAC, especially for those who are poor surgical candidates or with surgically unresectable tumors. When used in combination with other conventional antitumor interventions such as chemotherapy, EUS-guided RFA has been shown to potentially improve survival outcomes in patients with PDAC [97]. By shrinking tumor size, EUS-guided therapy has the added benefit of controlling local complications of malignancy bulk including pain and biliary obstruction, improving patient quality of life and providing a form of palliation for those who do not desire aggressive therapy [98].

## 9. Conclusions

EUS-guided local ablative therapies have shown promising technical feasibility, safety, and efficacy in the treatment of neuroendocrine and cystic neoplasms of the pancreas. In harnessing the antitumor effects of chemical toxicity and hyperthermia, EUS-guided chemical ablation and RFA balance targeted tissue necrosis with potential side effects on adjacent healthy parenchyma. These complications are mitigated by the benefits of real-time image guidance, close clinical follow-up, and careful selection of appropriate procedural candidates. To date, observational studies have demonstrated high clinical success of EUS-guided RFA and chemical ablation in the treatment of lesions with malignant potential, and emerging evidence highlights the growing technical and therapeutic scope of this minimally invasive intervention. Additional research is needed to determine the optimal procedural, demographic, and pathologic features that predict positive clinical outcomes.

## Figures and Tables

**Table 1:** Summary of Results for Selected Studies Utilizing EUS-Guided EA for PCN Therapy.

Author	Study Year	Ablative Strategy	Number of Treated Patients	Number of Treated Lesions			Efficacy on Follow-Up Imaging **, *n* (%)			Adverse Events, (*n)*
				MCN	IPMN	Other *	Incomplete Response	Partial Response	Complete Response	
Gan et al. [5]	2005	Ethanol (5–80%)	25	14	3	6	13 (56)	2 (9)	8 (35)	None
DeWitt et al. [59]	2009	Ethanol (80%)	25	10	10	5	13 (59)	0 (0)	9 (41)	Mild abdominal pain (7); Intra-cystic bleeding (1); Acute pancreatitis (1)
		Saline	17 ***	7	7	3	11 (79)	0 (0)	3 (21) ****	Mild abdominal pain (3)
DiMaio et al. [56]	2011	Ethanol (80%)	13	0	13	0	8 (62)	0 (0)	5 (38)	Mild abdominal pain (1)
Caillol et al. [24]	2012	Ethanol (99%)	13	14	0	0	2 (15)	0 (0)	11 (85)	None
Gómez et al. [57]	2016	Ethanol (80%)	23	4	15	4	11 (48)	10 (43)	2 (9)	Mild abdominal pain (1): Acute pancreatitis (1)
Park et al. [58]	2016	Ethanol (99%)	91	12	9	70	13 (14)	37 (41)	41 (45)	Mild abdominal pain (18); Fever without infection (8); Acute pancreatitis (3)

* The designation of “Other” includes patients treated in the above studies for cystic lesions that are non-neoplastic (i.e., pseudocysts), neoplastic without malignant potential (i.e., SCNs), or indeterminate based on pre-procedural analysis. Although the scope of this review focuses on the use of EUS-guided ablative procedures in the treatment of neoplastic cysts capable of malignant transformation (i.e., MCNs and IPMNs), this column is included in the table for the purpose of completeness. ** Complete response is defined as the radiographic absence of residual lesion on post-procedural imaging. Incomplete response is defined as either persistent or enlarged residual lesion on post-procedural imaging. If the study authors noted reduction in lesion size without resolution on post-procedural imaging, this is considered a partial response; if this was not recorded by the study authors, these lesions are categorized as incomplete response. *** Of the 17 patients initially treated with a session of saline lavage, 14 received a second follow-up session with ethanol lavage. **** All three patients with complete cyst resolution received initial saline lavage followed by a second session with ethanol lavage.

**Table 2:** Summary of Results for Selected Studies Utilizing EUS-Guided Intratumoral Drug Delivery for PCN Ablation.

Author	Study Year	Ablative Strategy	Number of Treated Patients	Number of Treated Lesions			Efficacy on Follow-Up Imaging **, *n* (%)			Adverse Events, (*n*)
				MCN	IPMN	Other *	Incomplete Response	Partial Response	Complete Response	
Oh et al. [7]	2008	Ethanol (88–99%) + Paclitaxel	14	2	0	12	1 (7)	2 (14)	11 (79)	Mild abdominal pain (1); Acute pancreatitis (1)
Oh et al. [61]	2009	Ethanol (99%) + Paclitaxel	10	3	0	7	2 (20)	2 (20)	6 (60)	Acute pancreatitis (1)
Oh et al. [6]	2011	Ethanol (99%) + Paclitaxel	52	9	0	43	12 (25)	6 (13)	29 (62)	Fever without infection (1); Mild abdominal pain (1); Acute pancreatitis (1); Splenic vein obliteration (1); Peri-cystic spillage (1)
Choi et al. [62]	2017	Ethanol (99%) + Paclitaxel	164	71	11	82	13 (8)	31 (20)	114 (72)	Fever without infection (1); Peri-cystic spillage (1); Intra-cystic bleeding (1); Acute pancreatitis (6); Pseudocyst formation (2); Abscess formation (2); Portal vein thrombosis (1); Splenic vein obliteration (1); MPD stricture (1)
Kim et al. [63]	2017	Ethanol (100%) or Ethanol (100%) + Paclitaxel	8 (Ethanol) 28 (Ethanol + Paclitaxel)	16	14	6	8 (24)	7 (20)	19 (56)	Mild abdominal pain (4); Acute pancreatitis (4); Intra-cystic bleeding (1)
Moyer et al. [64]	2017	Ethanol (80%) + Paclitaxel + Gemcitabine	18	9	27	3	3 (17)	4 (22)	11 (61)	Mild abdominal pain (4); Acute pancreatitis (1)
		Saline + Paclitaxel + Gemcitabine	21				4 (19)	3 (14)	14 (67)	None

* The designation of “Other” includes patients treated in the above studies for cystic lesions that are non-neoplastic (i.e., pseudocysts), neoplastic without malignant potential (i.e., SCNs), or indeterminate based on pre-procedural analysis. Although the scope of this review focuses on the use of EUS-guided ablative procedures in the treatment of neoplastic cysts capable of malignant transformation (i.e., MCNs and IPMNs), this column is included in the table for the purpose of completeness. ** Complete response is defined as the radiographic absence of residual lesion on post-procedural imaging. Incomplete response is defined as either persistent or enlarged residual lesion on post-procedural imaging. If the study authors noted reduction in lesion size without resolution on post-procedural imaging, this is considered a partial response; if this was not recorded by the study authors, these lesions are categorized as incomplete response.

**Table 3:** Summary of Results for Selected Studies Utilizing EUS-Guided RFA for PCN Therapy.

Author	Study Year	Number of Treated Patients	Number of Treated Lesions			Efficacy on Follow-Up Imaging **, *n* (%)			Adverse Events, (*n*)
			MCN	IPMN	Other *	Incomplete Response	Partial Response	Complete Response	
Pai et al. [11]	2015	6	4	1	1	0 (0)	4 (67)	2 (33)	Mild abdominal pain (2)
Barthet et al. [66]	2019	17	1	16	0	5 (29)	1 (6)	11 (65)	Jejunal perforation (1)
Oh et al. [68]	2021	13	0	0	13	5 (38)	8 (62)	0 (0)	Mild abdominal pain (1)
Younis et al. [67]	2022	5	1	4	0	1 (20)	1 (20)	3 (60)	Mild abdominal pain (2); Acute pancreatitis (1)

* The designation of “Other” includes patients treated in the above studies for cystic lesions that are non-neoplastic (i.e., pseudocysts), neoplastic without malignant potential (i.e., SCNs), or indeterminate based on pre-procedural analysis. Although the scope of this review focuses on the use of EUS-guided ablative procedures in the treatment of neoplastic cysts capable of malignant transformation (i.e., MCNs and IPMNs), this column is included in the table for the purpose of completeness. ** Complete response is defined as the radiographic absence of residual lesion on post-procedural imaging. Incomplete response is defined as either persistent or enlarged residual lesion on post-procedural imaging. If the study authors noted reduction in lesion size without resolution on post-procedural imaging, this is considered a partial response; if this was not recorded by the study authors, these lesions are categorized as incomplete response.

**Table 4:** Summary of Results for Selected Studies Utilizing EUS-Guided EA for pNET Therapy.

Author	Study Year	Ablative Strategy	Number of Treated Patients	Number of Treated Lesions		Efficacy on Follow-Up Imaging *, *n* (%)		Adverse Events, (*n*)
				Insulinoma	Non-Functional pNET	Incomplete Response	Complete Response	
Levy et al. [69]	2012	Ethanol (95–99%)	5	5	0	N/A **	N/A **	None
Park et al. [31]	2015	Ethanol (99%)	11	4	10	5 (38)	8 (62)	Mild abdominal pain (1); Acute pancreatitis (3); MPD stricture (1)
Choi et al. [70]	2018	Ethanol (99%) + Lipiodol	33	1	39	16 (40)	24 (60)	Acute pancreatitis (2)
Matsumoto et al. [71]	2020	Ethanol	5	0	5	1 (20)	4 (80)	None

* Complete response is defined as the radiographic absence of residual lesion on post-procedural imaging. Incomplete response is defined as either persistent or enlarged residual lesion on post-procedural imaging. ** No follow-up imaging was obtained to assess therapeutic efficacy, although 3 of 5 patients reported post-procedural resolution of hypoglycemic symptoms.

**Table 5:** Summary of Results for Selected Studies Utilizing EUS-Guided RFA for pNET Therapy.

Author	Study Year	Number of Treated Patients	Number of Treated Lesions		Efficacy on Follow-Up Imaging *, *n* (%)			Adverse Events, (*n*)
			Insulinoma	Non-Functional pNET	Incomplete Response	Partial Response	Complete Response	
Pai et al. [11]	2015	2	0	2	0 (0)	2 (100) **	0 (0)	None
Barthet et al. [66]	2019	12	0	14	2 (14)	0 (0)	12 (86)	Acute pancreatitis with necrosis and bacteremia (1); MPD stenosis (1)
Oleinikov et al. [74]	2019	18	9	18	1 (4)	0 (0)	26 (96)	Acute pancreatitis (2)
Marx et al. [75]	2022	7	7	0	0 (0)	0 (0)	6 (100)	Mild abdominal pain (1); Acute pancreatitis (2); Coagulation necrosis of the superior mesenteric vein (1); Retro-gastric collection resulting in death (1)
Marx et al. [76]	2022	27	0	27	0 (0)	2 (7)	25 (93)	Mild abdominal pain (3); Acute pancreatitis (4); Periprocedural bleeding (2); Pseudocyst formation (1); Pancreatic fistula formation (1); MPD stricture (1)
Figueiredo et al. [77]	2022	29 ***	13	10	2 (18)	3 (27)	6 (55)	Mild abdominal pain (4); Acute pancreatitis (3); MPD stenosis (1); Periprocedural bleeding (1); Gastric wall hematoma (1); Fever without infection (1)

* Complete response is defined as the radiographic absence of residual lesion on post-procedural imaging. Incomplete response is defined as either persistent or enlarged residual lesion on post-procedural imaging. If the study authors noted reduction in lesion size without resolution on post-procedural imaging, this is considered a partial response; if this was not recorded by the study authors, these lesions are categorized as incomplete response. ** Although complete lesion resolution was not observed, cross-sectional imaging revealed changes in tumor vascularity in one patient and central necrosis of the tumor in the other. *** This number of treated patients reflects the inclusion of one case of PDAC and 11 metastatic lesions in 6 patients who were subjected to EUS-guided RFA as part of the study population. These patients are not included in the columns displaying tumor efficacy but are included in the adverse events column.

## Data Availability

Not applicable.
